# A mixed-method comparison of physician-reported beliefs about and barriers to treatment with medications for opioid use disorder

**DOI:** 10.1186/s13011-020-00312-3

**Published:** 2020-09-14

**Authors:** Rebecca L. Haffajee, Barbara Andraka-Christou, Jeremy Attermann, Anna Cupito, Jessica Buche, Angela J. Beck

**Affiliations:** 1 From the Behavioral Health Workforce Research Center, University of Michigan, Ann Arbor, MI USA; 2 RAND Corporation, Boston, MA USA; 3 Department of Health Management and Policy, University of Michigan School of Public Health, Ann Arbor, MA USA; 4 Injury Prevention Center, Department of Emergency Medicine, University of Michigan Medical School, Ann Arbor, MI USA; 5 Department of Health Management & Informatics, University of Central Florida, Orlando, FL USA; 6 Department of Internal Medicine (Secondary Joint Appointment), University of Central Florida, Orlando, FL USA; 7 the National Council for Behavioral Health, Washington, D.C, USA; 8 Department of Health Behavior and Health Education, University of Michigan School of Public Health, Ann Arbor, MI USA

**Keywords:** Buprenorphine, Methadone, Naltrexone, Barriers, Physicians, Survey, Comparison, Referral, Dual diagnosis, Pregnant

## Abstract

**Background:**

Evidence demonstrates that medications for treating opioid use disorder (MOUD) —namely buprenorphine, methadone, and extended-release naltrexone—are effective at treating opioid use disorder (OUD) and reducing associated harms. However, MOUDs are heavily underutilized, largely due to the under-supply of providers trained and willing to prescribe the medications.

**Methods:**

To understand comparative beliefs about MOUD and barriers to MOUD, we conducted a mixed-methods study that involved focus group interviews and an online survey disseminated to a random group of licensed U.S. physicians, which oversampled physicians with a preexisting waiver to prescribe buprenorphine. Focus group results were analyzed using thematic analysis. Survey results were analyzed using descriptive and inferential statistical methods.

**Results:**

Study findings suggest that physicians have higher perceptions of efficacy for methadone and buprenorphine than for extended-release naltrexone, including for patients with co-occurring mental health disorders. Insurance obstacles, such as prior authorization requirements, were the most commonly cited barrier to prescribing buprenorphine and extended-release naltrexone. Regulatory barriers, such as the training required to obtain a federal waiver to prescribe buprenorphine, were not considered significant barriers by many physicians to prescribing buprenorphine and naltrexone in office-based settings. Nor did physicians perceive diversion to be a prominent barrier to prescribing buprenorphine. In focus groups, physicians identified financial, logistical, and workforce barriers—such as a lack of addiction treatment specialists—as additional barriers to prescribing medications to treat OUD.

**Conclusions:**

Additional education is needed for physicians regarding the comparative efficacy of different OUD medications. Governmental policies should mandate full insurance coverage of and prohibit prior authorization requirements for OUD medications.

## Background

### Opioid crisis

Recent indications suggest that the opioid-related overdose crisis is worsening in many regards, after claiming 47,600 lives in 2017 [1]. Between 2.3 and 6 million persons had an opioid use disorder (OUD) in 2017, only 20–40% of whom received addiction treatment [2]. Behavioral health workforce-related strategies to expand access to and delivery of evidence-based treatment for OUD are critical to reducing opioid-overdose risks and mitigating drug-related harms [3, 4].

### Treatment for opioid use disorder

Medications for OUD (MOUD), often in combination with behavioral therapy, are considered the gold standard for treating OUD [5]. Clinical trials have demonstrated that three MOUDs—methadone, buprenorphine, and extended-release naltrexone—reduce opioid use, overdose, and other adverse health outcomes, although methadone and buprenorphine appear to be more protective against overdose than extended-release naltrexone [6]. Methadone and buprenorphine treatment are associated with 53 and 37% reductions, respectively, in all-cause mortality among patients with OUD as compared to those receiving no MOUD in the 12 months following nonfatal overdose [7]. Buprenorphine availability starting in 2003 in Maryland also was associated with a 37% reduction in heroin overdose deaths [8].

### Access to treatment for opioid use disorder

Evidence suggests that MOUD access and treatment fall vastly below patient need [9, 10], owing in significant part to an under-supply of providers prescribing these medications [4, 11]. The number of Opioid Treatment Programs (OTPs), in which methadone is provided for OUD, has remained relatively flat over time [9, 10]. Many states have fewer than 10 OTPs, facilities that are scarce in rural areas [12–14]. In 2002, physicians became eligible to prescribe buprenorphine for OUD in non-specialty settings, provided they complete requisite training and obtain a buprenorphine waiver from the Drug Enforcement Administration (DEA) [15]. Although this regulatory change has expanded access to buprenorphine treatment for OUD, 44% of counties still lack a physician with a buprenorphine waiver, and only 3% of all primary care physicians nationwide are authorized to prescribe buprenorphine for OUD [12, 16]. Furthermore, substantial OUD treatment inequities exist along racial and ethnic lines, with Black patients having much lower odds of receiving buprenorphine for OUD than white patients [17–19]. Unlike methadone and buprenorphine, both opioid agonists, the newer extended-release naltrexone is an opioid antagonist and not a controlled substance; thus, it can be prescribed by any licensed prescriber.

Previous studies have identified numerous barriers to prescribing MOUD in office-based settings. The majority of such studies have focused on oral buprenorphine, finding salient barriers to include a lack of training for physicians in MOUD and addiction treatment, concerns about diversion, insurance barriers, and discomfort in treating patients with comorbid psychiatric conditions [11, 20–24]. Fewer studies have examined extended-release naltrexone; current research suggests that insurance-related factors, the requirement that patients are completely opioid-abstinent for 7 to 10 days prior to initiation, inadequate staffing, and limited education for prescribing physicians are key barriers to prescribing extended-release naltrexone [25–29]. Even though methadone for OUD cannot be prescribed outside of OTPs, office-based physicians can refer patients to these facilities for methadone treatment; but little is known about frequency of and barriers to this referral process. Furthermore, few studies have directly compared physician beliefs about efficacy and barriers across all three MOUDs [6, 27, 30].

In this mixed-methods study, we surveyed and conducted interviews with physicians to better understand and compare the facilitators and barriers they experience to prescribing (and referring, in the case of methadone) MOUDs. We hypothesized that prescriber beliefs about efficacy would be similarly positive for methadone and buprenorphine, with greater uncertainty expressed about the newer extended-release naltrexone, which has a less robust evidence base. We also hypothesized that perceived barriers to office-based buprenorphine prescribing would be most significant for physicians without a buprenorphine waiver and that patient opioid-abstinence would be a significant barrier to prescribing extended-release naltrexone treatment. However, we expected other barriers, like stigma [31] and insurance-related hurdles, to be consistent across MOUDs studied.

## Methods

To understand MOUD provision, barriers, and beliefs, we conducted a mixed-methods study that involved focus group interviews and an online survey disseminated to a random group of licensed U.S. physicians, which oversampled physicians with a preexisting waiver to prescribe buprenorphine. The study was one of concurrent data collection grounded in a complimentary perspective, with qualitative and quantitative data each contributing a different perspective to the phenomenon under study [32, 33].

The Health Sciences and Behavioral Sciences Institutional Review Board at the University of Michigan approved this study (reference number HUM00159099). The questions were informed by the previously discussed literature on prescriber-perceived efficacy of and barriers to MOUD treatment.

### Study design

We developed the survey using Qualtrics™ software and piloted it among physicians in four states in the Spring of 2017 (*n* = 53). See Additional file 2 for survey questions. We administered the final survey online in two waves from July 11—September 8, 2017, and from October 25—November 18, 2017. The survey was emailed to a nationally-representative random sample of 4010 physician prescribers, whose American Medical Association Masterfile contact and practice specialty information we purchased from Redi-Data. The sampled population, which included physicians practicing in all settings (including outpatient and inpatient), was divided among two groups: higher-frequency MOUD prescribers (*n* = 687, or physicians practicing addiction medicine and addiction psychiatry), and lower-frequency MOUD providers (*n* = 3313, or physicians practicing in general medicine specialties less likely to have regular exposure to MOUD prescribing). A total of 157 emails were returned as undeliverable, reducing the overall sampled population to 3853. Reminder emails were sent weekly and a $25 MasterCard gift card was offered an as incentive during the second wave of survey administration.

### Survey content

The survey examined provider-perceived barriers to and efficacy of the following MOUDs: oral buprenorphine, implantable buprenorphine, methadone, and depot injection extended-release naltrexone. We did not examine barriers to oral naltrexone prescribing, given its lack of efficacy for OUD due to low patient adherence [34] or to depot injection extended-release buprenorphine (Sublocade®) due to its recent Food and Drug Administration (FDA) approval [35]. Questions about Probuphine®, a diversion-resistant subdermal buprenorphine implant, were included in this study; however, not enough prescribers expressed familiarity with this formulation to assess specific barriers to its utilization.

Participants were asked to rate 17 different potential barriers to prescribing buprenorphine or extended-release naltrexone on a Likert scale, with answers ranging from “not a barrier at all” [1] to “strong barrier” [4]. If physicians indicated that they did not work with a particular medication (“N/A”), we removed these responses from the analysis. For buprenorphine, only those physicians who indicated they had a DEA waiver were asked about their perception of barriers to that MOUD. Because this survey primarily targeted office-based physicians, rather than those working in an OTP, questions about specific barriers to prescribing methadone were not included. All respondents were asked questions about the efficacy of each MOUD on a Likert scale that ranged from “strongly disagree,” [1] to “strongly agree” [5].

### Survey statistical analysis

We analyzed mean of the difference scores for key measures, defined as the differences across average scores reported along each potential barrier to buprenorphine and extended-release naltrexone and, separately, across average scores reported about MOUD efficacy beliefs using paired samples t-tests (with significance set at α = 0.05, two-tailed level). For all analyses, we also performed sub-analyses that involved independent samples t-tests to compare the responses of physicians who had a DEA waiver to prescribe buprenorphine for OUD to those who did not. For this sub-analysis, we first performed Levene’s Test for Equality of Variances to inform whether to assume equal variance between the groups; we assumed unequal variance if the test was significant at α = 0.05 level.^55^ A Bonferroni correction was performed to account for multiple testing for tests involving more than 7 comparisons.

### Qualitative data collection and analysis

To complement the survey data, we convened 3 virtual focus groups of prescribers to provide more in-depth information regarding MOUD provision in an office-based setting. Each focus group lasted approximately one hour and together they totaled 7 participants. We obtained a convenience sample of participants by leveraging the National Council for Behavioral Health’s communication channels, including email listserv, social media platform, and e-newsletter. Participants were drawn from mid-size and large cities across the country and were not necessarily MOUD prescribers. Focus group questions elicited barriers and facilitators to prescribing or referring patients to MOUD. We used thematic analysis methodology. Specifically, researchers created a codebook based on a preliminary review of transcripts. Then they independently coded transcripts, inductively identifying new potential codes using *Excel* and *NVivo 12* software [36]. They met to discuss discrepancies in coding, negotiating any differences. Researchers then reviewed codes for themes.

## Results

### Survey results

#### Survey respondent characteristics

Out of the 127 physicians included in the analysis, the majority of respondents were allopathic doctors (83%), male (59%), white (67%) and nonhispanic (93%). Additional File Table 1 lists respondent specialties. Respondents most frequently specialized in family medicine (34%), addiction medicine (25%), and anesthesiology (15%). These providers serve approximately 263 unique patients in an average month (*n* = 105).

Most respondents primarily practiced in outpatient primary care clinic settings (25%) or outpatient specialty clinics (14%), while only 5% practiced in an OTP. 35% reported that their practice facility was affiliated with a not-for-profit health center or hospital, 23% with an academic medical center, and 23% with a for-profit health center or hospital (Additional File Table 2).

45% of respondents indicated that they had a DEA buprenorphine waiver, although a small proportion were not currently using it (*n* = 6/104). 40% of physicians with DEA waivers could serve up to 100 patients. 76% of prescribers reported that they had not obtained the Risk Evaluation & Mitigation Strategy (REMS) certification to implant Probuphine® as treatment for OUD and did not plan to in the future (*n* = 77/101); 11% had the certification but were not currently implanting Probuphine® (*n* = 11/101); and no respondents had the certification and were implanting Probuphine.

45% of respondents indicated that no one in their practice currently prescribed extended-release naltrexone; only 22% indicated that they or someone else in their practice prescribed the medication. Only 16% of respondents answering the question indicated they often or always referred patients with OUD for methadone treatment, while 48% said they “sometimes” and 29% “never” did so.

#### Provider attitudes and beliefs about MOUD efficacy

Survey respondents had overall positive impressions of buprenorphine, extended-release naltrexone, and methadone for OUD treatment. However, there were some distinctions in beliefs about efficacy of the specific MOUDs. Table 1 depicts the comparison of respondent perceptions of the efficacy of buprenorphine and extended-release naltrexone. Respondents believed that buprenorphine, to a greater degree than extended-release naltrexone, decreases opioid cravings (paired t [37]=4.474, *p* < 0.001)., decrease the risk of fatal opioid-overdose (paired t [38]=3.413, *p* = 0.001), decreases return to opioid misuse (paired t [39]=2.078, *p* = 0.043), and works well in patients with co-occurring mental health disorders (paired t [39]=2.461, *p* = 0.017).

**Table 1:** Comparison of Provider-Perceived Efficacy of Buprenorphine vs. Extended-Release Naltrexone. Detailed Table Summary: Respondents believed that buprenorphine decreases opioid cravings more than extended-release naltrexone (paired t [37]=4.474, *p* < 0.001). Respondents believed that buprenorphine, to a greater degree than extended-release naltrexone, decreases the risk of fatal opioid-overdose (paired t [38]=3.413, *p* = 0.001), decreases return to opioid misuse (paired t [39]=2.078, *p* = 0.043), and works well in patients with co-occurring mental health disorders (paired t [39]=2.461, *p* = 0.017).

Perceptions	Buprenorphine		Naltrexone		t	df	Mean of the Difference	Cohen’s d	95% Confidence Interval		p
	n	Mean (SD)	n	Mean (SD)					Lower	Upper	
MOUD decreases risk of death from opioid overdose	52	4.327 (1.024)	52	3.769 (0.921)	3.413	51	0.558	0.755	0.230	0.885	0.001*
MOUD decreases cravings for opioids	53	4.491 (0.869)	53	3.566 (1.118)	4.474	52	0.925	0.924	0.510	1.339	< 0.001*
MOUD decreases rates of relapse	50	4.200 (1.030)	50	3.840 (0.792)	2.078	49	0.360	0.392	0.012	0.708	0.043*
MOUD works well in patients with co-occurring mental health disorders	50	4.220 (0.996)	50	3.780 (0.954)	2.461	49	0.440	0.451	0.081	0.799	0.017*
MOUD should be supplemented by mental health counseling	56	4.571 (0.806)	56	4.393 (0.846)	1.563	55	0.179	0.215	−0.050	0.408	0.124
MOUD should be supplemented by participation in peer support groups	57	4.298 (0.999)	57	4.193 (1.043)	0.973	56	0.105	0.103	−0.111	0.322	0.335
MOUD efficacy is improved by adding mental health counseling	56	4.518 (0.853)	56	4.429 (0.828)	0.962	55	0.089	0.106	−0.0979	0.2756	0.001*

*Notes: MOUD* medication for opioid use disorder. Questions asked about the MOUDs buprenorphine and extended-release naltrexone were compared in these results using paired samples t-tests (alpha = 0.05, two-tailed level). * indicates significance at the α = .05 level

Table 2 shows the comparison of provider-perceived efficacy of extended-release naltrexone and methadone to treat OUD. Respondents believed that methadone, to a greater degree than extended-release naltrexone, decreases opioid cravings (paired t [38]=3.759, *p* = 0.000), decreases risk of fatal opioid-overdose death (paired t [40]=2.349, *p* = 0.023), decreases return to opioid misuse (paired t [39]=2.780, *p* = 0.008), and works well in patients with co-occurring mental health disorders (paired t [39]=2.322, *p* = 0.024). When comparing physician perspectives about buprenorphine and methadone to treat OUD (Table 3), respondents believed that buprenorphine is slightly more effective than methadone in decreasing the risks of opioid-overdose death (paired t (67) = 2.147, *p* = 0.035).

**Table 2:** Comparison of Provider-Perceived Efficacy of Extended-Release Naltrexone vs. Methadone. Detailed Table Summary: Respondents believed that methadone, to a greater degree than extended-release naltrexone, decreases opioid cravings (paired t [38]=3.759, p = 0.000), decreases risk of fatal opioid-overdose death (paired t [40]=2.349, p = 0.023), decreases return to opioid misuse (paired t [39]=2.780, p = 0.008), and works well in patients with co-occurring mental health disorders (paired t [39]=2.322, p = 0.024)

Perceptions	Methadone		Naltrexone		t	df	Mean of the Difference	Cohen’s d	95% Confidence Interval		p
	n	Mean (SD)	n	Mean (SD)					Lower	Upper	
MOUD decreases risk of death from opioid overdose	51	4.078 (0.935)	51	3.745 (0.913)	−2.349	50	− 0.333	0.360	− 0.618	− 0.048	0.023*
MOUD decreases cravings for opioids	52	4.308 (0.853)	52	3.577 (1.126)	−3.759	51	−0.731	0.732	−1.121	− 0.341	0.000*
MOUD decreases rates of relapse	50	4.200 (0.808)	50	3.820 (0.774)	−2.780	49	−0.380	0.480	−0.655	− 0.105	0.008*
MOUD works well in patients with co-occurring mental health disorders	50	4.200 (0.857)	50	3.780 (0.954)	−2.322	49	−0.420	0.463	−0.784	− 0.057	0.024*
MOUD should be supplemented by mental health counseling	55	4.527 (0.790)	55	4.382 (0.850)	−1.343	54	−0.146	0.177	−0.363	0.072	0.185
MOUD should be supplemented by participation in peer support groups	56	4.304 (0.933)	56	4.179 (1.046)	−1.475	55	−0.125	0.126	−0.295	0.045	0.146
MOUD efficacy is improved by adding mental health counseling	55	4.309 (1.052)	55	4.418 (0.832)	0.973	54	0.109	0.115	−0.116	0.334	0.335

*Notes: MOUD* medication for opioid use disorder. Questions asked about the MOUDs extended-release naltrexone and methadone were compared using paired samples t- tests (alpha = 0.05, two-tailed level). * indicates significance at the α = .05 level

**Table 3:** Comparison of Provider-Perceived Efficacy of Buprenorphine vs. Methadone. Detailed Table Summary: When comparing physician perspectives about buprenorphine and methadone to treat OUD, respondents believed that buprenorphine is slightly more effective than methadone in decreasing the risks of opioid-overdose death (paired t (67) = 0.265, p = 0.035)

Perceptions	Buprenorphine		Methadone		t	df	Mean of the Difference	Cohen’s d	95% Confidence Interval		p
	n	Mean (SD)	n	Mean (SD)					Lower	Upper	
MOUD decreases risk of death from opioid overdose	68	4.294 (0.978)	68	4.029 (0.946)	2.147	67	0.265	0.275	0.019	0.511	0.035*
MOUD decreases cravings for opioids	66	4.349 (0.969)	66	4.227 (0.873)	1.070	65	0.121	0.132	−0.105	0.347	0.288
MOUD decreases rates of relapse	65	4.154 (1.004)	65	4.092 (0.931)	0.541	64	0.062	0.064	−0.166	0.289	0.590
MOUD works well in patients with co-occurring mental health disorders	63	4.016 (1.100)	63	4.032 (0.950)	−0.142	62	−0.016	0.016	−0.240	0.208	0.888
MOUD should be supplemented by mental health counseling	71	4.521 (0.954)	71	4.549 (0.842)	−0.363	70	−0.028	0.031	−0.183	0.127	0.718
MOUD should be supplemented by participation in peer support groups	69	4.333 (0.950)	69	4.391 (0.878)	−0.754	68	−0.058	0.063	−0.212	0.096	0.454
MOUD efficacy is improved by adding mental health counseling	69	4.551 (0.796)	69	4.391 (0.973)	1.469	68	0.159	0.180	−0.057	0.376	0.146

*Notes: MOUD* medication for opioid use disorder. Questions asked about the MOUDs buprenorphine and methadone were compared in these results using paired samples t-tests (alpha = 0.05, two-tailed level). * indicates significance at the α = .05 level

When comparing beliefs about MOUD efficacy among physicians with and without a DEA waiver, some significant differences emerged across medications. Waivered physicians agreed less strongly that buprenorphine is effective in treating opioid dependence in pregnant women, as compared to non-waivered physicians (paired t (67) = − 3.911, *p* = 0.000, Additional File Table 3). Waivered physicians believed that extended-release naltrexone treatment decreases the rate of return to opioid misuse to a greater degree than did non-waivered physicians (paired t [39]=2.143, *p* = 0.037, Additional File Table 4). Finally, waivered physicians, as compared to non-waivered physicians, believed less strongly that methadone decreases risk of opioid-overdose death (paired t (71) = − 3.097, *p* = 0.003, Additional File Table 5); decreases opioid cravings (paired t (70) = − 3.203, *p* = 0.002, Additional File Table 5), decreases rates of return to opioid misuse (paired t (62.573) = − 3.668, *p* = 0.001, Additional File 5), and is effective in treating OUD in pregnant women (paired t (65) = − 4.397, *p* < 0.001, Additional File Table 5).

#### Provider perceptions of barriers to office-based MOUD prescribing

**Fig. 1:**
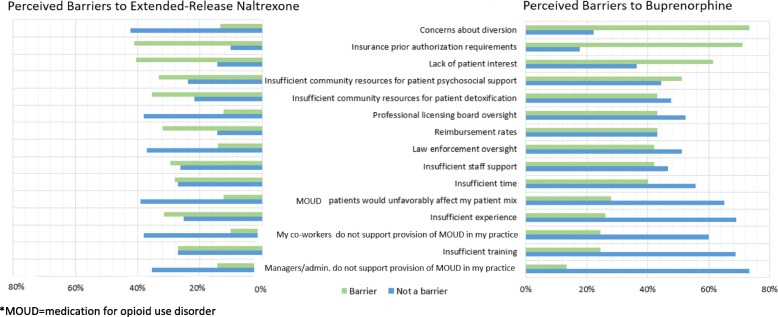
Perceived Barriers to Buprenorphine and Extended-Release Naltrexone. *Detailed Figure Summary*: The most common barrier to prescribing buprenorphine, according to buprenorphine-waivered physicians (*n* = 47 respondents), was insurance prior authorization requirements (22%), followed by insufficient staff support (16%). Lack of support by managers/administrators at the practice was most commonly identified as a non-barrier (73%), followed closely by insufficient training (69%). As with buprenorphine, a commonly cited barrier to prescribing extended-release naltrexone (*n* = 97 respondents) was insurance prior authorization requirements, as well as the lack of community resources for patient withdrawal management (each 16.5%). Concern about diversion was the most commonly identified non-barrier to prescribing extended-release naltrexone (42%).

Figure 1 summarizes prescriber beliefs about barriers to prescribing buprenorphine and extended-release naltrexone in office-based settings, using percentages to reflect the differing number of respondents for the two questions. The most common barrier to prescribing buprenorphine, according to DEA waivered physicians (*n* = 47 respondents), was insurance prior authorization requirements (22%), followed by insufficient staff support (16%). Lack of support by managers/administrators at the practice was most commonly identified as a non-barrier (73%), followed closely by insufficient training (69%). As with buprenorphine, a commonly cited barrier to prescribing extended-release naltrexone (*n* = 97 respondents) was insurance prior authorization requirements, as well as the lack of community resources for patient withdrawal management (each 16.5%). Concern about diversion was the most commonly identified non-barrier to prescribing extended-release naltrexone among all prescribers surveyed (42%). Table 4 shows the comparison of provider-perceived barriers to buprenorphine and extended-release naltrexone use for OUD. Respondents were statistically significantly more likely to be concerned about professional licensing board oversight (paired t [31]= 3.311, *p* = 0.002) for prescribing buprenorphine as compared to extended-release naltrexone.

**Table 4:** Comparison of Provider-Perceived Barriers to Buprenorphine vs. Extended-Release Naltrexone. Detailed Table Summary: Paired sample t-tests revealed statistically significantly different responses along only two barriers to prescribing buprenorphine versus extended-release naltrexone: concerns about diversion (paired t [33]=6.083, p = 0.000) and professional licensing board oversight (paired t [31]=3.311, p = 0.002)

Perceptions	Buprenorphine		Naltrexone		t	df	Mean of the Difference	Cohen’s d	95% Confidence Interval		p
	n	Mean (SD)	n	Mean (SD)					Lower	Upper	
Concerns about diversion	37	2.216 (1.084)	37	1.216 (0.630)	6.083	36	1.000	1.128	0.667	1.333	0.000*
Lack of patient interest	36	1.944 (1.068)	36	2.361 (1.046)	−1.838	35	− 0.417	0.394	− 0.877	0.044	0.075
Law enforcement oversight	37	1.757 (1.211)	27	1.270 (0.652)	2.991	36	0.486	0.501	0.157	0.816	0.005
Professional licensing board oversight	35	1.657 (1.056)	35	1.200 (0.584)	3.311	34	0.457	0.536	0.177	0.738	0.002*
MOUD patients would unfavorably affect my patient mix	33	1.394 (0.966)	33	1.212 (0.650)	1.359	32	0.182	0.221	−0.091	0.454	0.184
My co-workers do not support provision of MOUD in my practice	36	1.333 (0.894)	36	1.222 (0.591)	1.071	35	0.111	0.146	−0.099	0.322	0.291
Managers/ admin. do not support provision of MOUD in my practice	35	1.286 (0.869)	35	1.400 (0.946)	−0.702	34	−0.114	0.126	−0.445	0.217	0.487
Reimbursement rates for MOUD	33	1.879 (1.269)	33	2.424 (1.226)	−2.454	32	−0.545	0.437	−0.998	− 0.093	0.02
Insurance prior authorization requirements	37	2.378 (1.277)	37	2.784 (1.109)	−1.836	36	−0.405	0.339	−0.853	0.042	0.075
Insufficient training	35	1.371 (0.942)	35	1.571 (0.948)	−1.096	34	−0.200	0.212	−0.571	0.171	0.281
Insufficient time	36	1.778 (1.149)	36	1.611 (0.871)	0.784	35	0.167	0.164	−0.265	0.598	0.439
Insufficient staff support	35	1.914 (1.246)	35	1.771 (1.087)	0.776	34	0.143	0.122	−0.231	0.517	0.443
Insufficient experience	32	1.531 (1.016)	32	1.719 (0.991)	−0.882	31	−0.188	0.187	−0.621	0.246	0.385
Insufficient resources for patient psychosocial support within community or in my practice	35	1.857 (1.115)	35	1.657 (0.968)	1.125	34	0.200	0.192	−0.161	0.561	0.268
Insufficient resources for patient detoxification within the community or in my practice	34	1.706 (1.194)	34	1.971 (1.167)	−1.391	33	−0.265	0.224	−0.652	0.122	0.173

*Notes: MOUD* medication for opioid use disorder. Questions asked about the MOUDs buprenorphine and extended-release naltrexone were compared in these results using paired samples t-tests (alpha = 0.05, two-tailed level). * indicates significance once a Bonferroni Correction of α = .05/15 = .0033 was applied

Table 5 depicts the comparison of DEA-waivered and non-waivered prescribers’ perceptions of barriers to extended-release naltrexone. Waivered providers, as compared to non-waivered ones, were more concerned about the following with respect to extended-release naltrexone to treat OUD: insufficient training (paired t [40]=4.076, *p* = 0.000), insufficient time (paired t [38]=5.476, p = 0.000), insufficient staff support (paired t [38]=3.762, p = 0.000), insufficient experience (paired t [37]=5.175, p = 0.000\1), insufficient resources for patient psychosocial support (paired t [41]=5.855, p = 0.000), and insufficient resources for patient withdrawal management (paired t [41]=5.375, p = 0.000).

**Table 5:** Comparison of Perceived Efficacy of Extended-Release Naltrexone among SAMHSA-Waivered Physicians to Non-Waivered Physicians. Detailed Table Summary: Waivered providers, as compared to non-waivered ones, were more concerned about the following with respect to extended-release naltrexone to treat OUD: diversion (paired t (31.240) = 3.243, p = 0.003), insufficient training (paired t [40]=4.076, p = 0.000), insufficient time (paired t [38]=5.476, p = 0.000), insufficient staff support (paired t [38]=3.762, *p* = 0.000), insufficient experience (paired t [37]=5.175, *p* = 0.000), insufficient resources for patient psychosocial support (paired t [41]=5.855, *p* = 0.000), and insufficient resources for patient withdrawal management (paired t [41]=5.375, p = 0.000).

Perceptions	SAMHSA- waivered Physicians		Non-waivered Physicians		t	df	Mean Difference	Cohen’s d	95% Confidence Interval		p
	n	Mean (SD)	n	Mean (SD)					Lower	Upper	
Concerns about diversion^	23	1.83 (0.937)	31	1.129 (0.499)	3.243	31.240	0.697	0.934	0.259	1.135	0.003*
Lack of patient interest	21	2.429 (1.248)	32	2.375 (1.008)	0.172	51	0.054	0.048	−0.571	0.678	0.864
Law enforcement oversight^	19	1.790 (0.918)	31	1.226 (0.617)	2.369	28.063	0.564	0.721	0.076	1.051	0.025
Professional licensing board oversight^	18	2.111 (1.231)	31	1.161 (0.523)	3.114	20.614	0.950	1.004	0.315	1.585	0.005
Extended-release naltrexone patients would unfavorably affect my patient mix^	19	1.895 (1.100)	31	1.194 (0.654)	2.518	25.916	0.701	0.775	0.129	1.274	0.018
My co-workers do not support provision of extended-release naltrexone in my practice^	16	1.688 (0.946)	31	1.161 (0.523)	2.067	19.846	0.526	0.689	−0.005	1.057	0.052
Managers/administrators do not support provision of extended-release naltrexone in my practice^	18	2.167 (1.249)	30	1.333 (0.884)	2.483	27.318	0.833	0.771	0.145	1.522	0.019
Reimbursement rates for extended-release naltrexone	16	2.750 (1.125)	29	2.448 (1.298)	0.781	43	0.302	0.249	−0.477	1.081	0.439
Insurance prior authorization requirements^	18	2.944 (0.938)	32	2.750 (1.191)	0.637	42.622	0.194	0.181	−0.421	0.810	0.528
Insufficient training	21	2.762 (1.179)	31	1.548 (0.961)	4.076	50	1.214	1.129	0.616	1.812	0.000*
Insufficient time	22	2.818 (1.097)	31	1.419 (0.765)	5.476	51	1.399	1.479	0.886	1.912	0.000*
Insufficient staff support	22	2.773 (1.110)	31	1.645 (1.050)	3.762	51	1.128	1.044	0.526	1.729	0.000*
Insufficient experience	24	3.042 (1.160)	30	1.567 (0.935)	5.175	52	1.475	1.400	0.903	2.047	0.000*
Insufficient resources for patient psychosocial support within the community or in my practice	24	3.042 (0.955)	31	1.548 (0.925)	5.855	53	1.493	1.589	0.982	2.005	0.000*
Insufficient resources for patient detoxification within the community or in my practice	24	3.250 (0.989)	31	1.742 (1.064)	5.375	53	1.508	1.468	0.945	2.071	0.000*

*Notes*: Questions about the perceptions of DEA-waivered physicians with non-waivered physicians were compared in these results using independent samples t-test (α = .05, two-tailed level). * indicates significance once a Bonferroni Correction of α = .05/15 = .0033 was applied. ^ indicates that equal variances are not assumed

### Focus group results

Focus group participants were made up of 7 physicians (MD) across different states (PA, IL, FL, MO, ME, WA, CT), four of whom had a waiver to prescribe buprenorphine at the time of the focus group. Three identified as female and four as male. Participants in the focus groups provided more detail regarding 6 key themes identified in the coding process: MOUD efficacy, financial barriers to medications for OUD (provider- and client-side), treatment capacity, processes and procedures for treatment, provider competencies, and stigma. A list of selected quotations by theme is available in Table 6.

**Table 6:** Exemplar Quotations from Physician Focus Group. Detailed Table Summary: The table presents example quotes for six themes found from quantitative data analysis: 1) efficacy of medications for opioid use disorder, 2) financial barriers to medications for opioid use disorder, 3) treatment capacity, 4) processes and procedures for treatment, 5) provider competencies, and 6) stigma.

Theme 1: Efficacy of Medications for Opioid Use Disorder	
a.	“[T] he evidence base behind [extended-release naltrexone] right now is actually really limited. And it’s one of the things that makes me the most nervous when we talk about [MOUD], lumping them all together.”
b.	“I actually just put my last patient … with someone who has been through — he relapsed and was recommended to him by friends and family that he should not be on Suboxone just because he relapsed, and he’s been to detox four times in the last year. He’s been through multiple 28-day stays, all … abstinence-based. And, finally, came to the realization on his own that he did the best when he was on Suboxone. He had over a year of sobriety when he was on Suboxone, so he came back.”
c.	**“**We haven’t seen — at the one-year mark, we actually haven’t seen many positive outcomes [for patients receiving extended-release naltrexone]. We haven’t seen great retention in treatment, we haven’t seen a reduction in overdose, particularly at a year, we haven’t seen reduction in opioid use. Those first couple months, often, we will see it, but again, even in those first couple of months, the retention rates are really low compared to methadone and buprenorphine maintenance. And so, I’m not saying that I don’t think it should an option, but the same that I wouldn’t recommend a hypertension medication that has much worse outcomes as a first-line treatment, like I would only recommend [extended-release naltrexone] for people who are really aware that the outcomes are not nearly as good with Vivitrol as they are for buprenorphine or methadone maintenance”.
**Theme 2: Financial Barriers to Medications for Opioid Use Disorder (provider- and client-side)**	
a.	“And speaking to your question about coverage, even when folks had Medicaid or have Medicaid here, unless folks had a dual diagnosis, those programs feel very strongly that you cannot break even on the current reimbursement schedule. So, unless there’s another diagnosis — another major psychiatric diagnosis — in addition to the substance use disorder, or you have to be subsidizing the program from other parts of the services you provide, you can’t break even; even when folks are insured — is the perception here.”
b.	“For at least the State of Maine is, 40% of our folks who suffer right now from addiction, are uninsured. And so, that brings another layer of complexity of, you know, how are these people going to get care without necessarily dragging the program underground because of the lack of reimbursement with those patients. You know, where do we find that funding?”
c.	“I think the second thing is reimbursement. So, you know, when I first moved back to Illinois, buprenorphine, specifically, was not on Medicaid’s formulary. Which meant that, like, literally, every single month, my nurse — I had to have a full-time nurse assigned to just me to be able to start this program, just so she could spend all of her time filling out prior authorizations.”
**Theme 3: Treatment Capacity**	
a.	“I think a lot of people go to detox and then … it might be recommended that they move on to the next level of care, but there’s not capacity. So, then they’re sent home and (clinic staff) say, ‘Okay, we’ll put you on a wait list, and somebody will call you in the next month. And then you’ll hopefully get into treatment at that point.’ By the time that month comes around, most people have already relapsed.”
**Theme 4: Processes and Procedures for Treatment**	
a.	“Detox actually puts people at more harm for overdose than it does actually help them. Particularly if they’re not linked to the next level of care. And there are way more detox beds and way more detox capacity than there is access to actual continuation — continuing treatment. So, this is a system that’s sort of designed to fail, in my mind.”
b.	“We should be putting a lot more resources into recovery-oriented systems that are going to be — continuing with the long-term, and less resources into detox for opioids specifically. For alcohol, it’s obviously very necessary.”
c.	“I think that the real problem comes down to sort of the way that primary care is reimbursed right now. And that, you know, the way that things have been structured, we get these very very short visits. And particularly, again, in under-served settings like, you know — you’re seeing uninsured patients as well, where you get no payment as well. So, you have to be able to balance all of that.”
**Theme 5: Provider Competencies**	
a.	“And then, I think the knowledge — feeling uncomfortable with just an eight-hour course to take to obtain the waiver. A lot of people felt that would be insufficient to actually have a good comfort. And that’s despite us expressing that there will be (inaudible 0:28:59) support with addiction. Psychiatrists. There’s still a lot of trepidation. And just trying to fit that in with their regular panel patients.”
b.	“People just don’t feel that well-versed in either how to talk to people about it, or if someone is screening positive, what am I supposed to do next? And, you know, if they don’t have behavioral health support within their clinic setting, then it’s really hard because they often don’t have the skills or the knowledge to be able to provide all of that behavioral support.”
**Theme 6: Stigma**	
a.	“Definitely stigma — it’s shocking to hear some providers say, “Well, I don’t want that patient withdrawing in the waiting room beside my two-year-old, you know, toddler that I’m going to see, you know, in the afternoon,” or whatever. There were just different excuses for — but a big part of it was there was this undercurrent of stigma.”
b.	“And I think there’s a lot of stigma against methadone sort of everywhere. There’s some stigma against Suboxone or buprenorphine in Baltimore, but people, when I came back to Chicago, just never even really heard of it as a treatment option unless they were people who had lived in other states.”
c.	“I think physicians have big practices, and they don’t want 200 opioid addicts to be in their waiting room a lot, I think.”

#### MOUD efficacy

With respect to MOUD efficacy (Theme 1 in Table 6), focus group participants noted disparities in the evidence base for different MOUDs. According to one focus group participant, “[t] he evidence base behind [extended-release naltrexone] right now is actually really limited. And it’s one of the things that makes me the most nervous when we talk about [MOUD], lumping them all together.” This perception was borne out by survey results that indicated greater belief in the efficacy of methadone and buprenorphine as compared to extended-release naltrexone.

#### Logistical and financial barriers

The focus groups also highlighted financial and logistical barriers to providing MOUD treatment (Themes 2, 3, and 4 in Table 6). For example, participants raised concerns about the staff time and cost of acquiring necessary continuing education to provide MOUD, as well as the difficulties in ensuring a practice’s financial sustainability across the diverse MOUD billing codes and reimbursement rates. One provider stated that running an OUD program would lose money for their practice (quote 1a, Table 6). Providers also noted difficulties in establishing necessary workflows for providing MOUD, particularly in the context of multidisciplinary teams (quote 3c, Table 6). Finally, many focus group participants cited the lack of addiction treatment providers within their community as a significant barrier to patients (quote 2a, Table 6).

#### Provider perceptions and stigma

Focus group participants also emphasized the negative or uninformed perceptions associated with training for and treating patients with OUD and expressed a reluctance to treat what they perceived to be a potentially challenging population (Themes 5 and 6 in Table 6). Several providers expressed concern with their knowledge and the training demands to treat patients with OUD (quotes 4a and 4b, Table 6). One provider raised concerns about practices, particularly large ones, attracting a patient population dominated by persons with OUD (quote 5c, Table 6). Providers did emphasize the importance of psychosocial support as a component of OUD addiction treatment services, in addition to MOUD (quote 5a, Table 6). Another participant said that providers do not feel comfortable talking to patients who screen positive for OUD, often lack the knowledge to provide behavioral health support, and do not have access to on-site support from counselors or psychologists/psychiatrists (quote 4a, Table 6).

## Discussion

Our mixed methods study compared physician prescriber perceptions of efficacy and barriers to OUD treatment across three MOUDs using both focus group data and survey data. In terms of barriers, we focused on prescribing in office-based treatment settings (i.e., naltrexone and buprenorphine prescribing), but also asked about referral to methadone clinics. We compared responses from those physicians with and without a DEA waiver to prescribe buprenorphine. The survey data complemented by qualitative responses provides new and timely information on MOUD treatment beliefs and challenges.

Our study found that insurance barriers, specifically prior authorization requirements, were the most commonly cited barrier to buprenorphine and extended-release naltrexone prescribing. While few other studies have explored barriers to extended-release naltrexone prescribing [26, 27], partly owing to its relatively recent FDA-approval for OUD, other studies have likewise found that insurance requirements are a strong barrier to buprenorphine prescribing [20, 42, 43]. By confirming results from these other studies, our study lends further support to the need for federal and state governments to intervene in decreasing insurance barriers to MOUD. For example, federal and state authorities should strengthen enforcement of parity laws and sanction violations related to inequitable treatment limitations applied to OUD treatment. These barriers may be quantitative (e.g., the number of days of treatment coverage) or non-quantitative (e.g., fail first requirements or prior authorization requirements). Furthermore, given Medicaid’s important role in ensuring OUD treatment [44], states should expand Medicaid and eliminate prior authorization requirements for buprenorphine and extended-release naltrexone covered by Medicaid programs.

Interestingly, we found that regulatory barriers were ranked lower than other barriers to buprenorphine prescribing, despite the existence of relatively unique buprenorphine prescribing regulations, such as patient limits and special education requirements. This could be due to our sampling strategy, which oversampled physicians with a preexisting waiver to prescribe buprenorphine (approximately 40% of our sample) even though only approximately 2% of U.S. physicians have a waiver [16]. Individuals who do not view buprenorphine prescribing regulations as a salient barrier may have self-selected into the group that has already obtained a waiver. Future studies should further examine the perception of regulatory barriers among a representative sample of physicians who have not yet obtained a waiver. Some previous studies may have oversampled physicians without a waiver; and physicians without a waiver may overestimate the difficulty of adhering to patient limits, completing special education requirements, and applying to SAMHSA for a waiver. Eliminating the waiver to obtain buprenorphine could address perceived barriers to buprenorphine prescribing among certain prescribers [3]. Alternatively, over time, physicians may find it easier to meet regulatory requirements, especially as the availability of online education courses has increased. Also, the institutions in which physicians work may be increasing their support of buprenorphine prescribing over time, thereby giving physicians time and funds to complete the waiver process. Future studies should examine the impact of educational availability and institutional support on perceptions of regulatory barriers.

Our study found higher perceptions of efficacy in treating OUD for methadone and buprenorphine than for extended-release naltrexone. This discrepancy may be explained by greater awareness of methadone and buprenorphine (which were FDA-approved prior to extended-release naltrexone) and fewer published studies about extended-release naltrexone—a point noted by focus group participants. Recently, some studies have found similar efficacy between buprenorphine and extended-release naltrexone for OUD [30, 45], while another more recent study found lower efficacy of extended-release naltrexone in terms of overdose protection [6]; but these studies were unavailable or very recent when we surveyed participants. Additionally, many physicians in our sample have limited experiential knowledge of naltrexone if no one in their practice is prescribing it. Our interpretation is limited by the fact that we did not ask survey participants whether they are currently prescribing extended-release naltrexone. Finally, our participants may feel that extended-release naltrexone is less effective for patients who are not yet opioid-abstinent or are unwilling or unable to withdraw from opioids, even though the medication may be effective for patients in other practices who have already completed the withdrawal management process.

Participants believed that buprenorphine is slightly more effective than methadone at preventing opioid overdose, among other measures of efficacy, although the scholarly literature suggests that methadone and buprenorphine efficacy is comparable, with a literature review finding that methadone is slightly more effective at retaining patients in treatment than buprenorphine [46, 47]. Retention is critical, as longer retention with either buprenorphine or methadone is associated with lower rates of opioid overdose and opioid-related acute care use [6]. In a randomized control multisite trial, 74% of patients randomized to methadone completed treatment at 24 weeks, as compared to 46% of those randomized to buprenorphine/naloxone [48]. Higher doses of buprenorphine or methadone are associated with longer retention [47, 48]. Respondents may simply have been less familiar with the literature about methadone and with real-world effectiveness of methadone, since they cannot prescribe it in office-based settings. Additionally, patients who seek OUD treatment in office-based settings may have stronger pre-existing preferences for buprenorphine than for methadone [49, 50], making providers in such settings less likely to seek out education about or to refer patients to methadone treatment.

Although little has been written about the appropriateness of prescribing methadone, buprenorphine, or extended-release naltrexone for individuals with co-occurring mental health disorders, our participants believed that methadone and buprenorphine are more appropriate than extended-release naltrexone for dual diagnosis patients. Possibly participants are aware that depression is an adverse event associated with extended-release naltrexone in about 10% of patients [39]. The literature on extended-release naltrexone’s efficacy in dual diagnosis patients may also be less developed because of its novelty in treating OUD and/or because participants are more risk averse to prescribing it. Furthermore, since patients beginning extended-release naltrexone treatment must be opioid abstinent for at least seven days, healthcare practitioners may feel that this hurdle is too difficult for individuals with dual diagnosis to overcome. Given the correlation between OUD and mental health disorders [40], significantly more research is needed regarding the effectiveness of MOUD for individuals with dual diagnosis and barriers to prescribing MOUD for this population.

Non-waivered participants believed methadone and buprenorphine are highly effective for pregnant women, but waivered participants as compared to non-waivered participants had more negative beliefs about the effectiveness of each medication for this population. Possibly the waivered survey participants do not routinely treat pregnant women for OUD (though we did not explicitly ask about this) and are thus more risk averse to using MOUD for pregnant women. Both methadone and buprenorphine are effective for pregnant women with OUD [37, 38]. Therefore, education about methadone’s and buprenorphine’s efficacy in pregnant women should be part of courses for obtaining a SAMHSA waiver, especially in light of increasing rates of OUD in pregnant women and of neonatal withdrawal syndrome [41].

Participants were significantly less likely to identify regulatory concerns (e.g., diversion and licensing board oversight) as barriers to extended-release naltrexone prescribing than to buprenorphine prescribing. This result is not surprising, since misuse or diversion of extended-release naltrexone is unlikely, given its office-based administration and lack of a psychoactive ingredient. Nevertheless, even for buprenorphine, participants did not, on average, believe diversion and licensing board oversight were strong barriers to prescribing. However, our study oversampled waivered physicians; and physicians who have sought and obtained a waiver may as a group be less likely to have diversion or oversight concerns than physicians who have not sought and obtained a waiver. Nevertheless, real-world experiences of those actually waivered to prescribe buprenorphine are important to the extent they reflect that diversion is not a high concern with this medication, to refute longstanding stigma.

No participants were implanting Probuphine®, likely reflecting the novelty of the medication. Even though we included questions about Probuphine® in our survey, due to sample size limitations, not enough data was gathered to assess specific barriers to its utilization. Future studies should explore the extent to which the REMS certification serves as a barrier to prescribing Probuphine®, as well as barriers associated with the need to stabilize patients on oral buprenorphine prior to Probuphine® administration. Additionally, future studies should examine barriers to Sublocade® prescribing.

Our study has several limitations. We generated a new survey that has not been validated, and some of our results (e.g., in terms of buprenorphine diversion concerns) may be statistically significant but are unlikely to be clinically significant. Our survey response rate was small relative to the population sampled, likely because the incentives offered were small and because this population may be experiencing survey fatigue, so our results may not be generalizable to all physician prescribers. Our final sampled population over-represented physicians with a buprenorphine waiver, so our results may represent a bias in favor of MOUD treatment and more moderate perceptions of barriers. However, this may suggest that once prescribers become waivered and prescribe MOUDs, that the actual barriers to this treatment for OUD may be less substantial than previously perceived. Finally, we did not ascertain whether respondents were currently prescribing extended-release naltrexone, a potential variable related to perceptions of efficacy and barriers.

Our findings suggest that there is room for improvement in OUD treatment education. For example, less is known about newer medications—especially implantable and injectable buprenorphine—and these are areas for further training. Also, MOUD treatment in pregnant women was not well understood among participants and warrants additional training. Finally, persistent insurance barriers to MOUD prescribing, including prior authorization, continue to merit attention and parity enforcement from regulators. Public payers can act as market leaders in generously covering MOUD,so that prescribers and patients do not perceive these as significant obstacles to effective care.

## Conclusion

Our study compared physician beliefs about the efficacy of and barriers to three types of medications for OUD treatment. We found that physicians reported insurance barriers as more common than either regulations or diversion concerns for both oral buprenorphine and extended-release naltrexone. Physicians in our sample believed that oral buprenorphine and methadone have greater efficacy than extended-release naltrexone in treating OUD. Physicians also believed that buprenorphine and methadone are superior treatments for patients with dual diagnoses – an underexamined issue in previous literature. Also, physicians in our sample believed that buprenorphine was more effective than methadone at treating OUD – a conclusion that may result in too few referrals to methadone treatment. Additional education for physicians about comparative efficacy of OUD treatment is needed.

## Supplementary information

**Additional file 1 Appendix A Table 1:** Survey Respondent Specialties. Appendix A Table 2: Survey Respondent Practice Setting. **Appendix A Table 3:** Comparison of Perceived Efficacy of Buprenorphine Among SAMHSA-Waivered Physicians to Non-Waivered Physicians. **Appendix A Table 4:** Comparison of Perceived Efficacy of Extended-Release Naltrexone Among SAMHSA-Waivered Physicians vs. Non-Waivered Physicians. **Appendix A Table 5:** Comparison of Perceived Efficacy of Methadone Among SAMHSA-Waivered Physicians vs. Non-Waivered Physicians.

**Additional file 2.** Appendix B: Survey Instrument.

## Data Availability

Materials are available from the first author upon request. To protect the confidentiality of study participants, survey data and interview data is not available.

## Abbreviations

MOUD: Medication for opioid use disorder
OUD: Opioid use disorder
