# Supplementary material for: A mixed-method comparison of physician-reported beliefs about and barriers to treatment with medications for opioid use disorder
Source: Subst Abuse Treat Prev Policy. 2020 Sep 14;15:69. doi: 10.1186/s13011-020-00312-3 (PMC7491096; doi:10.1186/s13011-020-00312-3)
Supplement: Supplementary file 1 — Additional file 1 Appendix A Table 1: Survey Respondent Specialties. Appendix A Table 2: Survey Respondent Practice Setting. Appendix A Table 3: Comparison of Perceived Efficacy of Buprenorphine Among SAMHSA-Waivered Physicians to Non-Waivered Physicians. Appendix A Table 4: Comparison of Perceived Efficacy of Extended-Release Naltrexone Among SAMHSA-Waivered Physicians vs. Non-Waivered Physicians. Appendix A Table 5: Comparison of Perceived Efficacy of Methadone Among SAMHSA-Waivered Physicians vs. Non-Waivered Physicians. [file 13011_2020_312_MOESM1_ESM.docx]

**Appendix A: Additional Tables**

**Appendix Table 1: Survey Respondent Specialties**

| Specialty | Number (%) |
| --- | --- |
| *Low Frequency MOUD Prescribers* | |
| Family Medicine | 41 (34%) |
| Psychiatry (general) | 16 (13%) |
| Anesthesiology | 18 (15%) |
| Internal Medicine | 4 (3%) |
| Geriatric Medicine | 2 (2%) |
| Hospitalist | 2 (2%) |
| Adolescent Medicine | 1 (1%) |
| Emergency Medicine | 1 (1%) |
| Oncology | 1 (1%) |
| Preventive Medicine & Public Health | 1 (1%) |
| *High Frequency MOUD Prescribers* | |
| Addiction Psychiatry | 2 (2%) |
| Addiction Medicine | 30 (25%) |

**Appendix Table 2: Survey Respondent Practice Setting**

| Practice Setting | Count (%) |
| --- | --- |
| Outpatient Primary Care Clinic (Private Practice or Academic) | 37 (25%) |
| Outpatient Specialty Clinic (Private Practice or Academic) | 21 (14%) |
| General Hospital (but not in ER) | 19 (13%) |
| Community Health Center/Public Health Clinic | 14 (9%) |
| Substance Abuse Treatment Facility (Outpatient) | 13 (8.6%) |
| Other | 8 (5%) |
| Certified Opioid Treatment Program | 7 (4.6%) |
| Substance Abuse Treatment Facility (Inpatient) | 7 (4.6%) |
| Emergency Room | 6 (4%) |
| Outpatient Surgery Center | 4 (3%) |
| Veterans Administration Hospital | 4 (3%) |
| Psychiatric Hospital (but not in ER) | 3 (2%) |
| Veterans Administration Clinic | 3 (2%) |
| Long-Term Acute Care Hospital (but not in ER) | 2 (1.3%) |
| Diagnostic Testing Facility | 1 (0.7%) |
| Pain Management Clinic | 1 (0.7%) |
| Urgent Care Facility | 1 (0.7%) |
| Total | 151 (100%) |

*Note*: Percentages add to more than 100% due to rounding.

| **Appendix Table 3: Comparison of Perceived Efficacy of Buprenorphine Among DEA-Waivered Physicians to Non-Waivered Physicians** | | | | | | | | | | | |
| --- | --- | --- | --- | --- | --- | --- | --- | --- | --- | --- | --- |
| Perceptions | DEA- waivered Physicians | | Non-waivered Physicians | | t | df | Mean Difference | Cohen’s d | 95% Confidence Interval | | p |
|  | n | Mean(SD) | n | Mean(SD) |  |  |  |  | Lower | Upper |  |
| Patients treated with buprenorphine are difficult to manage | 30 | 3.067 (1.230) | 40 | 2.375 (1.234) | 2.324 | 68 | 0.692 | 0.562 | 0.098 | 1.286 | 0.023 |
| Buprenorphine decreases risk of death from opioid overdose | 37 | 4.081 (0.722) | 40 | 4.525 (1.062) | -2.128 | 75 | -0.444 | 0.489 | -0.860 | -0.028 | 0.037 |
| Buprenorphine treatment decreases cravings for opioids | 36 | 4.111 (0.747) | 40 | 4.550 (1.085) | -2.031 | 74 | -0.439 | 0.471 | -0.869 | -0.008 | 0.046 |
| Buprenorphine treatment decreases rates of relapse | 35 | 3.857 (0.845) | 39 | 4.436 (0.995) | -2.681 | 72 | -0.579 | 0.627 | -1.009 | -0.149 | 0.009 |
| Buprenorphine treatment works well in patients with co-occurring mental health disorders | 33 | 3.667 (0.990) | 39 | 4.333 (1.084) | -2.705 | 70 | -0.667 | 0.642 | -1.158 | -0.175 | 0.009 |
| Buprenorphine should be supplemented by mental health counseling | 39 | 4.564 (0.852) | 40 | 4.525 (0.987) | 0.188 | 77 | 0.039 | 0.042 | -0.374 | 0.453 | 0.851 |
| Buprenorphine treatment should be supplemented by participation in peer support groups^ | 39 | 4.462 (0.643) | 40 | 4.275 (1.109) | 0.917 | 62.835 | 0.187 | 0.206 | -0.220 | 0.593 | 0.362 |
| Buprenorphine treatment efficacy is improved by adding mental health counseling^ | 39 | 4.615 (0.544) | 40 | 4.475 (0.933) | 0.819 | 63.030 | 0.140 | 0.183 | -0.203 | 0.484 | 0.418 |
| Buprenorphine treatment is effective for treating opioid dependence in pregnant women | 31 | 3.581 (0.886) | 38 | 4.421 (0.889) | -3.911 | 67 | -0.840 | 0.946 | -1.269 | -0.412 | 0.000* |

*Notes:* Questions about the perceptions of DEA-waivered physicians with non-waivered physicians were compared in these results using independent samples t-test (α=.05, two-tailed level). * indicates significance once a Bonferroni Correction of α=.05/9 =.00556 has been applied. ^ indicates that equal variances are not assumed.

| **Appendix Table 4: Comparison of Perceived Efficacy of Extended-Release Naltrexone Among DEA-Waivered Physicians vs. Non-Waivered Physicians** | | | | | | | | | | | |
| --- | --- | --- | --- | --- | --- | --- | --- | --- | --- | --- | --- |
| Perceptions | DEA- waivered Physicians | | Non-waivered Physicians | | t | df | Mean Difference | Cohen’s d | 95% Confidence Interval | | p |
|  | n | Mean(SD) | n | Mean(SD) |  |  |  |  | Lower | Upper |  |
| XR-NTX decreases risk of death from opioid overdose | 21 | 3.476  (0.873) | 31 | 3.968 (0.912) | -1.970 | 50 | -0.492 | 0.551 | -1.001 | 0.017 | 0.058 |
| XR-NTX decreases cravings for opioids | 21 | 3.619  (0.973) | 32 | 3.531  (1.218) | 0.277 | 51 | 0.088 | 0.080 | -0.548 | 0.724 | 0.783 |
| XR-NTX decreases rates of relapse | 21 | 3.571  (0.746) | 30 | 4.033  (0.765) | -2.143 | 49 | -0.462 | 0.611 | -0.895 | -0.029 | 0.037* |
| XR-NTX works well in patients with co-occurring mental health disorders | 20 | 3.500  (1.051) | 31 | 3.968  (0.836) | -1.762 | 49 | -0.468 | 0.493 | -1.001 | 0.066 | 0.084 |
| XR-NTX should be supplemented by mental health counseling | 24 | 4.250  (0.989) | 32 | 4.500  (0.718) | -1.096 | 54 | -0.250 | 0.289 | -0.707 | 0.207 | 0.278 |
| XR-NTX should be supplemented by participation in peer support groups | 24 | 4.167  (0.963) | 33 | 4.212  (1.111) | -0.161 | 55 | -0.045 | 0.043 | -0.611 | 0.520 | 0.873 |
| XR-NTX efficacy is improved by adding mental health counseling | 23 | 4.435  (0.728) | 33 | 4.424  (0.902) | 0.046 | 54 | 0.011 | 0.013 | -0.445 | 0.466 | 0.963 |

*Notes:* XR-NTX=extended-release naltrexone treatment. Questions about the perceptions of DEA-waivered physicians with non-waivered physicians were compared in these results using independent samples t-test (α=.05, two-tailed level). * indicates significance once a Bonferroni Correction of α=.05/7 =.0071 has been applied. ^ indicates that equal variances are not assumed.

| **Appendix Table 5: Comparison of Perceived Efficacy of Methadone Among DEA-Waivered Physicians vs. Non-Waivered Physicians** | | | | | | | | | | | |
| --- | --- | --- | --- | --- | --- | --- | --- | --- | --- | --- | --- |
| Perceptions | DEA- waivered Physicians | | Non-waivered Physicians | | t | df | Mean Difference | Cohen’s d | 95% Confidence Interval | | p |
|  | n | Mean(SD) | n | Mean(SD) |  |  |  |  | Lower | Upper |  |
| Methadone decreases risk of death from opioid overdose. | 38 | 3.658 (0.878) | 35 | 4.314 (0.932) | -3.097 | 71 | -0.656 | 0.725 | -1.079 | -0.234 | 0.003* |
| Methadone treatment decreases cravings for opioids. | 38 | 3.868 (0.844) | 34 | 4.500 (0.826) | -3.203 | 70 | -0.632 | 0.502 | -1.025 | -0.238 | 0.002* |
| Methadone treatment decreases rates of relapse. | 38 | 3.658 (1.047) | 35 | 4.400 (0.651) | -3.668 | 62.573 | -0.742 | 0.851 | -1.147 | -0.338 | 0.001* |
| Methadone treatment works well in patients with co-occurring mental health disorders | 37 | 3.730 (0.962) | 34 | 4.294 (0.799) | -2.677 | 69 | -0.564 | 0.638 | -0.985 | -0.144 | 0.009 |
| Methadone should be supplemented by mental health counseling. | 38 | 4.737 (0.446) | 35 | 4.343 (1.083) | 2.001 | 44.465 | 0.394 | 0.476 | -0.003 | 0.791 | 0.051 |
| Methadone treatment should be supplemented by participation in peer support groups. | 38 | 4.553 (0.602) | 34 | 4.235 (1.075) | 1.522 | 50.557 | 0.317 | 9.736 | -0.101 | 0.736 | 0.134 |
| Methadone is effective for treating OUD in pregnant women. | 33 | 3.485 (0.972) | 34 | 4.471 (0.861) | -4.397 | 65 | -0.986 | 1.074 | -1.433 | -0.538 | 0.000* |
| Methadone treatment efficacy is improved by adding mental health counseling. | 36 | 4.500 (0.845) | 35 | 4.257 (1.094) | 1.049 | 69 | 0.243 | 0.249 | -0.219 | 0.705 | 0.298 |
| Most patients who legally obtain methadone for addiction divert it. | 35 | 2.257 (1.010) | 33 | 2.303 (1.262) | -.166 | 66 | -0.046 | 0.040 | -0.598 | 0.506 | 0.869 |
| Individuals who purchase methadone illicitly do so primarily to become sober or prevent withdrawal symptoms. | 37 | 2.946 (1.129) | 32 | 3.375 (0.907) | -1.722 | 67 | -0.429 | 0.419 | -0.926 | 0.068 | 0.090 |
| Individuals who purchase methadone illicitly do so primarily to experience euphoria. | 36 | 3.139 (1.125) | 32 | 2.563 (0.948) | 2.269 | 66 | 0.576 | 0.554 | 0.069 | 1.084 | 0.027 |

*Notes*: Questions about the perceptions of DEA-waivered physicians with non-waivered physicians were compared in these results using independent samples t-test (α=.05, two-tailed level). * indicates significance once a Bonferroni Correction of α=.05/11 =.0045 has been applied.
