# Supplementary material for: A mixed-method comparison of physician-reported beliefs about and barriers to treatment with medications for opioid use disorder
Source: Subst Abuse Treat Prev Policy. 2020 Sep 14;15:69. doi: 10.1186/s13011-020-00312-3 (PMC7491096; doi:10.1186/s13011-020-00312-3)
Supplement: Supplementary file 2 — Additional file 2. Appendix B: Survey Instrument. [file 13011_2020_312_MOESM2_ESM.docx]

Appendix B: Survey Instrument

**Scopes of Practice Issues Related to Medication-Assisted Treatment**
  You are invited to take this online survey because you are a physician potentially eligible to prescribe medication-assisted treatment (MAT) medications. We at the Behavioral Health Workforce Research Center (BHWRC), a partnership between the University of Michigan School of Public Health and the National Council for Behavioral Health (National Council), would like to better understand the prescriber perceptions of and barriers to utilizing MAT. Your input is very important to us and we appreciate your time and feedback!
  This online survey will take approximately 10-15 minutes to complete. The survey does not need to be completed in one sitting. If you need to exit the survey, you will be able to start it again at the same computer.

 Your participation in this research study is voluntary. You may choose not to participate. If you decide to participate in this survey, you may withdraw at any time. If you decide not to participate, or if you withdraw, you will not be penalized.   To protect your confidentiality, this survey will not ask you to provide any information that will personally identify you, such as your name, email address, or Social Security number.  All survey results will be stored securely at the University of Michigan. Only members of the BHWRC and the National Council will use this survey information. Survey responses will not be released to employers or anyone other than research staff of the BHWRC.   For your participation in this project, **you may choose to enter into a random drawing for one of six $50 MasterCard Gift Cards**. Participation in the drawing is voluntary; to enter, you will be prompted to provide your contact information at the end of the survey. Your contact information provided will not be connected to any responses you give.   This research has been reviewed according to the University of Michigan Institutional Review Board (IRB) procedures for research involving human subjects. Participation in this survey implies consent. If you have any questions about the research study, please contact: Megan Dormond, MPH, Project Manager, Behavioral Health Workforce Research Center at the National Council at BHWRC@thenationalcouncil.org.

End of Block: Background

Start of Block: Demographic

**Section I. Demographics** This section will ask about your demographic information.

What best describes your gender?

- Female
- Male
- Other

What best describes your race? (Check all that apply)

- American Indian or Alaskan Native
- Asian
- Black/African American
- Hispanic/Latino
- Native Hawaiian/Pacific Islander
- White/Caucasian
- Other (Please Specify) ________________________________________________

Are you Hispanic, Latino/a, or of Spanish origin? (One or more categories may be selected.)

- ⊗No
- Yes, Mexican, Mexican American, Chicano/a
- Yes, Puerto Rican
- Yes, Cuban
- Yes, other (please specify) ________________________________________________
- ⊗Prefer not to answer

End of Block: Demographic

Start of Block: Practice Type

**Section II. Practice Characteristics**
 The next set of questions will ask you to provide information about your practice.

What best describes your licensure?

- Medical Doctor (MD)
- Doctor of Osteopathy (DO)
- Doctor of Podiatric Medicine (DPM)
- Other (Please specify) ________________________________________________

What best describes your primary area of medical practice?

- Addiction Medicine
- Adolescent Medicine
- Allergy & Immunology
- Anesthesiology
- Cardiology
- Cardiac & Thoracic Surgery
- Critical Care
- Colon & Rectal Surgery
- Dermatology
- Emergency Medicine
- Family Medicine
- General Practice
- Gastroenterology
- General Surgery
- Geriatric Medicine
- Hospice & Palliative Medicine
- Hospitalist
- Interventional Radiology
- Internal Medicine
- Medical Pediatrics
- Neurology
- Neurosurgery
- Nuclear Medicine
- Obstetrics/Gynecology
- Oncology
- Ophthalmology
- Other (Please Specify) ________________________________________________

What best describes your secondary area of medical practice?

- Internal Medicine
- Orthopedic Surgery
- Otolaryngology/ENT
- Pain Medicine
- Pathology
- Pediatrics
- Physical Medicine & Rehabilitation
- Plastic Surgery
- Preventive Medicine
- Psychiatry/Mental Health
- Pulmonary Disease
- Radiology
- Radiation Oncology
- Rheumatology
- Sports Medicine
- Urology
- Vascular Surgery
- Other (Please Specify) ________________________________________________

What best describes your primary practice setting (where you see the majority of patients)? (Select all that apply)

- Certified Opioid Treatment Program
- Community Health Center/Public Health Clinic
- Diagnostic Testing Facility
- Emergency Room
- General Hospital (but not in ER)
- Psychiatric Hospital (but not in ER)
- Long-Term Acute Care Hospital (but not in ER)
- Outpatient Specialty Clinic (Private Practice or Academic)
- Outpatient Primary Care Clinic (Private Practice or Academic)
- Outpatient Surgery Center
- Veterans Administration Clinic
- Veterans Administration Hospital
- Pain Management Clinic
- Rehabilitation Hospital
- Retail Medicine Clinic (CVS Minute Clinic, Walgreens Health Clinic, Clinic at Wal-Mart)
- Substance Abuse Treatment Facility (Outpatient)
- Substance Abuse Treatment Facility (Inpatient)
- Urgent Care Facility
- Other (Please Specify) ________________________________________________

Please mark all that describe your primary practice facility affiliations. (Select all that apply)

- My practice is affiliated with or within a for-profit health center or hospital
- My practice is affiliated with or within a not-for-profit health center or hospital
- My practice is affiliated with an academic medical center
- My practice is affiliated with a detoxification center
- Other ________________________________________________

How many years have you been practicing in your primary field?

- 1-2
- 3-5
- 6-10
- 11-15
- 16-20
- 21-25
- 26-30
- 31-35
- 36-40
- 41+

In what zip code is your practice?

________________________________________________________________

________________________________________________________________

________________________________________________________________

________________________________________________________________

________________________________________________________________

On average approximately how many unique patients do you serve in an average month?

________________________________________________________________

Roughly what percent of your patient payment revenue comes from the following sources? (Categories should sum to 100%)

Medicare : _______

Medicaid : _______

Dual Eligibles (Both Medicare and Medicaid) : _______

Private insurance : _______

Self-pay only : _______

Charity/Sliding Scale Pay : _______

IHS : _______

Veterans Administration : _______

Other (Please Specify) : _______

I don’t know : _______

Total : ________

End of Block: Practice Type

Start of Block: Screening for SUD/Managing OUD

S**ection III. Screening and Managing Substance Use Disorders**
 The next set of questions will ask you to provide information about screening and managing Substance Use and Opioid Use Disorders.

What percentage of new patients do you screen for Substance Use Disorder (SUD)?

- 0%
- 1-25%
- 26-50%
- 51-75%
- 76-99%
- 100%

For what percentage of your patients do you treat or manage Opioid Use Disorder treatment?

- 0%
- 1-25%
- 26-50%
- 51-75%
- 76-99%
- 100%

Skip To: End of Block If For what percentage of your patients do you treat or manage Opioid Use Disorder treatment? = 0%

How do you treat or manage Opioid Use Disorder within your practice?

|  | Never | Sometimes | Often | Always | N/A | I don’t work directly with patients receiving OUD |
| --- | --- | --- | --- | --- | --- | --- |
| I refer patients with OUD to specialist behavioral health physicians (e.g. psychiatrists or addictionologists) |  |  |  |  |  |  |
| I refer patients with OUD to non-specialists physicians (e.g. primary care physicians) |  |  |  |  |  |  |
| I refer patients with OUD to physician assistants or nurse practitioners |  |  |  |  |  |  |
| I refer patients with OUD to mental health therapists (non-physicians) |  |  |  |  |  |  |
| I refer patients with OUD to certified Opioid Treatment Programs |  |  |  |  |  |  |
| I refer patients with OUD to residential treatment centers |  |  |  |  |  |  |
| I collaborate with physicians in treating patients with OUD |  |  |  |  |  |  |
| I collaborate with mental health therapists (non-physicians) in treating patients with OUD |  |  |  |  |  |  |

How do you monitor Opioid Use Disorder?

|  | Never | Sometimes | Often | Always | N/A | I don’t work directly with patients receiving OUD |
| --- | --- | --- | --- | --- | --- | --- |
| Patient self-report of cravings |  |  |  |  |  |  |
| Patient self-report of substance use |  |  |  |  |  |  |
| Observation of physical function/appearance |  |  |  |  |  |  |
| Urine or other drug screening |  |  |  |  |  |  |
| Reports from other treatment providers |  |  |  |  |  |  |
| Use validated mental health screening tools (such as the Patient Health Questionnaire) |  |  |  |  |  |  |
| Use validated SUD screening tools, such as the Opioid Risk Tool (ORT), Screener and Opioid Assessment for Patients with Pain (SOAPP), or Current Opioid Misuse Measure (COMM) |  |  |  |  |  |  |

Do you have access to (i.e., are you enrolled in) your state prescription drug monitoring program (PDMP)?

- Yes
- No
- I do not know

Skip To: Q16 If Do you have access to (i.e., are you enrolled in) your state prescription drug monitoring program... = Yes

Skip To: End of Block If Do you have access to (i.e., are you enrolled in) your state prescription drug monitoring program... = No

Skip To: End of Block If Do you have access to (i.e., are you enrolled in) your state prescription drug

When prescribing controlled substances, how often do you use your state's prescription drug monitoring program for the following reasons:

|  | Never | Sometimes | Often | Always | N/A |
| --- | --- | --- | --- | --- | --- |
| To identify whether the patient has a potential SUD |  |  |  |  |  |
| To Identify any potential negative drug interactions prior, I.e. Drug reconciliation (e.g. Prevent patients from concurrently receiving benzos and opioids) |  |  |  |  |  |
| To identify whether the patient may be doctor shopping |  |  |  |  |  |

End of Block: Screening for SUD/Managing OUD

Start of Block: General Prescribing/ Dispensing

**Section IV. Prescribing and Dispensing Behavior**
 The next set of questions will ask you to provide information about prescribing and dispensing behaviors.

How much formal education did you receive regarding medication-assisted treatment for Opioid Use Disorder?

|  | 0 Hours | Less than 2 hours | 3-5 hours | 6-8 hours | 9-11 hours | 12-14 hours | 15+ hours | N/A |
| --- | --- | --- | --- | --- | --- | --- | --- | --- |
| Undergraduate |  |  |  |  |  |  |  |  |
| Medical School (or other graduate program) |  |  |  |  |  |  |  |  |
| Residency |  |  |  |  |  |  |  |  |
| Fellowship |  |  |  |  |  |  |  |  |
| Continuing Medical Education |  |  |  |  |  |  |  |  |

End of Block: General Prescribing/ Dispensing

Start of Block: Buprenorphine

**Section V. Buprenorphine**
 The next set of questions will ask you to provide information about Buprenorphine.

Have you obtained a waiver from the Substance Abuse and Mental Health Services Administration (SAMHSA) to prescribe buprenorphine (or buprenorphine-naloxone) for OUD?

- Yes, and I currently prescribe it for OUD
- Yes, but I do not currently prescribe it for OUD
- No, but I plan to obtain the waiver in the future
- No, and I do not plan to obtain the waiver in the future

Skip To: Q25 If Have you obtained a waiver from the Substance Abuse and Mental Health Services Administration (SA... = No, but I plan to obtain the waiver in the future

Skip To: Q25 If Have you obtained a waiver from the Substance Abuse and Mental Health Services Administration (SA... = No, and I do not plan to obtain the waiver in the future

How many patients does your SAMHSA waiver allow you to treat?

- Up to 30
- Up to 100
- Up to 275

Do you supervise any Nurse Practitioners or Physician Assistants who prescribe buprenorphine?

- Yes. I supervise NPs or PAs.
- No.
- No, but I am planning to

How long have you had a SAMHSA waiver? (Years)

▼ 1 (1) ... 20 (20)

How long have you been prescribing buprenorphine for OUD maintenance treatment? (Years)

▼ 1 (1) ... 20 (20)

What types of insurance do you accept from patients for treatment with buprenorphine? (mark all that apply)

- I DO NOT accept any commercial or public insurance for any visits in my practice (including for non-buprenorphine treatment)
- I DO NOT accept any commercial or public insurance for buprenorphine treatment visits, but I DO accept commercial or public insurance for other types of treatment
- I accept commercial health insurance for treatment with buprenorphine
- I accept Medicaid for treatment with buprenorphine
- I accept Medicare for treatment with buprenorphine
- I have a sliding scale payment option for treatment with buprenorphine
- Other (Please Specify) ________________________________________________

To your knowledge, do any other physicians in your practice have a SAMHSA waiver for prescribing buprenorphine treatment?

- Yes
- No
- I do not know
- There are no other physicians within my practice

Display This Question:

If Have you obtained a waiver from the Substance Abuse and Mental Health Services Administration (SA... = Yes, and I currently prescribe it for OUD

Or Have you obtained a waiver from the Substance Abuse and Mental Health Services Administration (SA... = Yes, but I do not currently prescribe it for OUD

Please indicate how much of a barrier each of the following circumstances is to providing buprenorphine treatment within your practice:

|  | Not a barrier at all | Somewhat of a barrier | Moderate Barrier | Strong barrier | Not applicable | I don’t work directly with patients receiving buprenorphine |
| --- | --- | --- | --- | --- | --- | --- |
| Concerns about diversion |  |  |  |  |  |  |
| Lack of patient interest |  |  |  |  |  |  |
| Law enforcement oversight |  |  |  |  |  |  |
| Professional licensing board oversight |  |  |  |  |  |  |
| Buprenorphine treatment patients would unfavorably affect my patient mix |  |  |  |  |  |  |
| My co-workers do not support provision of buprenorphine treatment in my practice |  |  |  |  |  |  |
| Managers/administrators do not support provision of buprenorphine treatment in my practice |  |  |  |  |  |  |
| Reimbursement rates for buprenorphine treatment |  |  |  |  |  |  |
| Insurance prior authorization requirements |  |  |  |  |  |  |
| Patient limits to prescribing buprenorphine |  |  |  |  |  |  |
| Record keeping and reporting requirements for buprenorphine treatment |  |  |  |  |  |  |
| Insufficient training |  |  |  |  |  |  |
| Insufficient time |  |  |  |  |  |  |
| Insufficient staff support |  |  |  |  |  |  |
| Insufficient experience |  |  |  |  |  |  |
| Insufficient resources for patient psychosocial support within the community or in my practice |  |  |  |  |  |  |
| Insufficient resources for patient detoxification within the community or in my practice |  |  |  |  |  |  |
| Other (Please Specify) |  |  |  |  |  |  |

How strongly do you agree with the following statements about buprenorphine (or buprenorphine-naloxone) treatment?

|  | Strongly Disagree | Disagree | Neither Agree nor Disagree | Agree | Strongly Agree | N/A | I don’t work directly with patients receiving buprenorphine |
| --- | --- | --- | --- | --- | --- | --- | --- |
| Patients treated with buprenorphine are difficult to manage |  |  |  |  |  |  |  |
| Buprenorphine treatment decreases risk of death from opioid overdose. |  |  |  |  |  |  |  |
| Buprenorphine treatment decreases cravings for opioids. |  |  |  |  |  |  |  |
| Buprenorphine treatment decreases rates of relapse. |  |  |  |  |  |  |  |
| Buprenorphine treatment works well in patients with co-occurring mental health disorders |  |  |  |  |  |  |  |
| Buprenorphine should be supplemented by mental health counseling. |  |  |  |  |  |  |  |
| Buprenorphine treatment should be supplemented by participation in peer support groups. |  |  |  |  |  |  |  |
| Buprenorphine treatment efficacy is improved by adding mental health counseling. |  |  |  |  |  |  |  |
| Buprenorphine treatment is effective for treating opioid dependence in pregnant women. |  |  |  |  |  |  |  |

End of Block: Buprenorphine

Start of Block: Probuphine

**Section VI. Probuphine**
 The next set of questions will ask you to provide information about Probuphine.

Have you obtained the Risk Evaluation & Mitigation Strategy (REMS) certification to implant Probuphine?

- Yes, and I currently implant Probuphine
- Yes, but I do not currently implant Probuphine
- No, but I prescribe Probuphine and collaborate with a physician who implants Probuphine
- No, but I plan to obtain the REMS certification to implant Probuphine
- No, and I do not plan to obtain the REMS certification to implant Probuphine

To your knowledge, have any other physicians within your practice obtained Risk Evaluation & Mitigation Strategy (REMS) certification to implant Probuphine?

- Yes
- No
- I don’t know
- There are no other physicians within my practice

Display This Question:

If Have you obtained the Risk Evaluation & Mitigation Strategy (REMS) certification to implant Probu... = Yes, and I currently implant Probuphine

And Have you obtained the Risk Evaluation & Mitigation Strategy (REMS) certification to implant Probu... = Yes, but I do not currently implant Probuphine

And Have you obtained the Risk Evaluation & Mitigation Strategy (REMS) certification to implant Probu... = No, but I prescribe Probuphine and collaborate with a physician who implants Probuphine

Please indicate how much of a barrier the following factors are to prescribing Probuphine in your practice.

|  | Not a barrier | Somewhat of a barrier | Moderate Barrier | Strong barrier | Not applicable | I don’t work directly with patients receiving Probuphine |
| --- | --- | --- | --- | --- | --- | --- |
| Concerns about Probuphine diversion |  |  |  |  |  |  |
| Concerns about patients removing Probuphine implant from their arm |  |  |  |  |  |  |
| Lack of patient interest |  |  |  |  |  |  |
| Law enforcement oversight |  |  |  |  |  |  |
| Professional licensing board oversight |  |  |  |  |  |  |
| Probuphine treatment patients would unfavorably affect my patient mix |  |  |  |  |  |  |
| My co-workers do not support provision of Probuphine treatment in my practice |  |  |  |  |  |  |
| Managers/administrators do not support provision of Probuphine treatment in my practice |  |  |  |  |  |  |
| Reimbursement rates for Probuphine treatment |  |  |  |  |  |  |
| Insurance prior authorization requirements |  |  |  |  |  |  |
| Patient limits to prescribing Probuphine |  |  |  |  |  |  |
| Record keeping and reporting requirements for Probuphine treatment |  |  |  |  |  |  |
| The SAMHSA waiver course |  |  |  |  |  |  |
| The REMS certification process |  |  |  |  |  |  |
| Insufficient training |  |  |  |  |  |  |
| Insufficient time |  |  |  |  |  |  |
| Insufficient staff support |  |  |  |  |  |  |
| Insufficient experience |  |  |  |  |  |  |
| Insufficient community resources for patient psychosocial support |  |  |  |  |  |  |
| Insufficient community resources for patient detoxification |  |  |  |  |  |  |
| Other (Please Specify) |  |  |  |  |  |  |

How strongly do you agree with the following statements about Probuphine treatment?

|  | Strongly Disagree | Disagree | Neither Agree nor Disagree | Agree | Strongly Agree | N/A | I don’t work directly with patients receiving Probuphine |
| --- | --- | --- | --- | --- | --- | --- | --- |
| Probuphine treatment decreases risk of death from opioid overdose. |  |  |  |  |  |  |  |
| Probuphine treatment decreases cravings for opioids. |  |  |  |  |  |  |  |
| Probuphine treatment decreases rates of relapse. |  |  |  |  |  |  |  |
| Probuphine treatment works well in patients with co-occurring mental health disorders |  |  |  |  |  |  |  |
| Probuphine should be supplemented by mental health counseling. |  |  |  |  |  |  |  |
| Probuphine treatment should be supplemented by participation in peer support groups. |  |  |  |  |  |  |  |
| Probuphine treatment efficacy is improved by adding mental health counseling. |  |  |  |  |  |  |  |

End of Block: Probuphine

Start of Block: Vivitrol/Naloxone/Methadone

**Section VII. Vivitrol/Naloxone/Methadone**
 The next set of questions will ask you to provide information about Vivitrol/Naloxone/Methadone.

To your knowledge, do any other physicians, NPs or PAs within your practice prescribe Vivitrol (extended-release naltrexone) for OUD?

- Yes
- No
- I don’t know
- There are no other physicians, NPs, or PAs within my practice

Please indicate how much of a barrier the following factors are to prescribing Vivitrol in your practice.

|  | Not a barrier | Somewhat of a barrier | Moderate Barrier | Strong barrier | Not applicable | I don’t work directly with patients receiving Vivitrol |
| --- | --- | --- | --- | --- | --- | --- |
| Concerns about diversion |  |  |  |  |  |  |
| Lack of patient interest |  |  |  |  |  |  |
| Law enforcement oversight |  |  |  |  |  |  |
| Professional licensing board oversight |  |  |  |  |  |  |
| Vivitrol treatment patients would unfavorably affect my patient mix |  |  |  |  |  |  |
| My co-workers do not support provision of Vivitrol treatment in my practice |  |  |  |  |  |  |
| Managers/administrators do not support provision of Vivitrol treatment in my practice |  |  |  |  |  |  |
| Reimbursement rates for Vivitrol treatment |  |  |  |  |  |  |
| Insurance prior authorization requirements |  |  |  |  |  |  |
| Insufficient training |  |  |  |  |  |  |
| Insufficient time |  |  |  |  |  |  |
| Insufficient staff support |  |  |  |  |  |  |
| Insufficient experience |  |  |  |  |  |  |
| Insufficient community resources for patient psychosocial support |  |  |  |  |  |  |
| Insufficient community resources for patient detoxification |  |  |  |  |  |  |
| Other (Please Specify) |  |  |  |  |  |  |

How strongly do you agree with the following statements about Vivitrol treatment?

|  | Strongly Disagree | Disagree | Neither Agree nor Disagree | Agree | Strongly Agree | N/A | I don’t work directly with patients receiving Vivitrol |
| --- | --- | --- | --- | --- | --- | --- | --- |
| Vivitrol treatment decreases risk of death from opioid overdose. |  |  |  |  |  |  |  |
| Vivitrol treatment decreases cravings for opioids. |  |  |  |  |  |  |  |
| Vivitrol treatment decreases rates of relapse. |  |  |  |  |  |  |  |
| Vivitrol treatment works well in patients with co-occurring mental health disorders |  |  |  |  |  |  |  |
| Vivitrol should be supplemented by mental health counseling. |  |  |  |  |  |  |  |
| Vivitrol treatment should be supplemented by participation in peer support groups. |  |  |  |  |  |  |  |
| Vivitrol treatment efficacy is improved by adding mental health counseling. |  |  |  |  |  |  |  |

Have often do you refer patients to methadone treatment for OUD

- Never
- Sometimes
- Often
- Always
- NA- because there is no access to methadone treatment in my community
- NA-other ________________________________________________

Skip To: Q41 If Have often do you refer patients to methadone treatment for OUD = NA-other

Skip To: Q41 If Condition: NA-other Is Not Empty. Skip To: Please indicate whether any of the fo....

How strongly do you agree with the following statements about methadone treatment for opioid use disorder?

|  | Strongly Disagree | Disagree | Neither Agree nor Disagree | Agree | Strongly Agree | N/A | I don’t work directly with patients receiving methadone |
| --- | --- | --- | --- | --- | --- | --- | --- |
| methadone treatment decreases risk of death from opioid overdose. |  |  |  |  |  |  |  |
| methadone treatment decreases cravings for opioids. |  |  |  |  |  |  |  |
| methadone treatment decreases rates of relapse. |  |  |  |  |  |  |  |
| methadone treatment works well in patients with co-occurring mental health disorders |  |  |  |  |  |  |  |
| methadone should be supplemented by mental health counseling. |  |  |  |  |  |  |  |
| methadone treatment should be supplemented by participation in peer support groups. |  |  |  |  |  |  |  |
| methadone is effective for treating OUD in pregnant women |  |  |  |  |  |  |  |
| methadone treatment efficacy is improved by adding mental health counseling |  |  |  |  |  |  |  |
| Most patients who legally obtain methadone for addiction treatment divert it |  |  |  |  |  |  |  |
| Individuals who purchase methadone illicitly do so primarily to become sober or prevent withdrawal symptoms |  |  |  |  |  |  |  |
| Individuals who purchase methadone illicitly do so primarily to experience euphoria (“get high”) |  |  |  |  |  |  |  |

Please indicate whether any of the following factors increase or decrease your likelihood of prescribing or referring patients to specific medications for opioid dependence maintenance/management.

|  | Factor changes likelihood of prescribing/referring **methadone** | | Factor changes likelihood of prescribing/referring **buprenorphine** | | Factor changes likelihood of prescribing/referring **Vivitrol** | |
| --- | --- | --- | --- | --- | --- | --- |
|  | Increases | Decreases | Increases | Decreases | Increases | Decreases |
| ⊗Patient has severe opioid dependence. |  |  |  |  |  |  |
| ⊗Patient has low treatment motivation. |  |  |  |  |  |  |
| ⊗Patient is an injection drug user. |  |  |  |  |  |  |
| ⊗Patient has co-occurring mental health disorders. |  |  |  |  |  |  |
| ⊗Patient is currently involved in criminal justice system. |  |  |  |  |  |  |
| ⊗Patient is relatively young |  |  |  |  |  |  |
| ⊗Patient has long history of opioid use disorder |  |  |  |  |  |  |
| ⊗Patient has a stable family/support network |  |  |  |  |  |  |
| ⊗Patient has previously purchased buprenorphine or methadone illicitly |  |  |  |  |  |  |
| ⊗Patient has previously diverted buprenorphine or methadone |  |  |  |  |  |  |
| ⊗Patient has health insurance |  |  |  |  |  |  |
| ⊗Patient has a history of compliance with treatment protocol |  |  |  |  |  |  |
| ⊗Patient has regular transportation access |  |  |  |  |  |  |
| ⊗Patient is deeply engaged in 12-step philosophy |  |  |  |  |  |  |
| ⊗Patient is pregnant |  |  |  |  |  |  |
| ⊗Patient suffers from chronic pain |  |  |  |  |  |  |

Please answer the following questions regarding naloxone.

|  | Never | Rarely | Often | Always | NA |
| --- | --- | --- | --- | --- | --- |
| I prescribe naloxone to patients at risk for opioid use disorder. |  |  |  |  |  |
| I discuss naloxone with patients at risk for opioid use disorder. |  |  |  |  |  |
| I prescribe naloxone to families of patients at risk for opioid use disorder. |  |  |  |  |  |
| I discuss naloxone with families of patients at risk for opioid use disorder. |  |  |  |  |  |

End of Block: Vivitrol/Naloxone/Methadone

Start of Block: Further Information

Please include any other comments or additional information you would like to share.

________________________________________________________________

________________________________________________________________

________________________________________________________________

________________________________________________________________

________________________________________________________________

End of Block: Further Information

Start of Block: Random Drawing

Thank you for completing our survey. If you would like to be in included in the drawing for one of six $50 gift cards, please leave your name and email address below:

- Full Name (1) ________________________________________________
- Email Address (2) ________________________________________________

End of Block: Random Drawing
